# Supplementary material for: Content-rich biological network constructed by mining PubMed abstracts
Source: BMC Bioinformatics. 2004 Oct 8;5:147. doi: 10.1186/1471-2105-5-147 (PMC528731; doi:10.1186/1471-2105-5-147)
Supplement: Additional File 2 — The original results of the above study (non-essential files are deleted to keep the file size under the limit set by BMC bioinformatics). [file 1471-2105-5-147-S2.bz2 › chilibotAdditionalFile2/dip20/162ID8612709E567/html/ACVR1B_ACVR2.html]

 


 **ACVR1B** and **ACVR2** 
  
Found 46 abstracts in PubMed, retrieved 20.  
 

 What does Google say? 
 PDF only 
| .edu only 

---

**Interactive relationship** (e.g. stimulation, inhibition, etc)

**Inhibitory relationship**- A kinase deleted version of ALK4  [ **ACVR1B** ]  can form an inactive complex with activin and ActRII  [ **ACVR2** ]  IIB and thereby acts in a dominant negative manner to block activin signaling.  Ref: 12665502 J Biol Chem< MedlineTA>J Biol Chem,
**Neutral relationship**- Isoform analysis of the major functional type I activin receptor  [ **ACVR2** ]  alk4  [ **ACVR1B** ]  revealed the full length transcript SKR2  [ **ACVR1B** ]  1 and transcripts for the partially truncated proteins SKR2  [ **ACVR1B** ]  2 and SKR2  [ **ACVR1B** ]  3 in normal thyroids and goiters.  Ref: 11069203 Horm Metab Res, 2000

**Non-interactive relationship** (e.g. studied together, co-existance, homology, etc.)

- Activins, like other members of the TGF beta superfamily, initiate signaling by assembling a complex of two types of transmembrane serine threonine receptor kinases classified as type II ActRII  [ **ACVR2** ]  or ActRIIB and type I ALK4  [ **ACVR1B** ] .  Ref: 12665502 J Biol Chem< MedlineTA>J Biol Chem, 2000
- The Activin receptor  [ **ACVR2** ]  ALK4  [ **ACVR1B** ]  is also able to mediate Nodal signaling but only in the presence of Cripto, with which it can also interact directly.  Ref: 11485994 Genes Dev, 2001
- Expression of dominant negative mutants of various receptors indicated that ALK4  [ **ACVR1B** ] , and either ActRIIA  [ **ACVR2** ]  or ActRIIB, function as type I and type II receptors for Nodal, respectively.  Ref: 11952836 Genes Cells, 2002
- **ACVR1B**  ALK4  [ **ACVR1B** ] , activin receptor  [ **ACVR2** ]  type 1B gene mutations in pancreatic carcinoma.  Ref: 11248065 Proc Natl Acad Sci U S A, 2001
- This was accompanied by strong upregulation of the activin receptor  [ **ACVR2** ]  ALK 4  [ **ACVR1B** ]  ActR IB  [ **ACVR1B** ]  and redistribution of the TGF beta responsive ALK 5 TbetaR I.  Ref: 11472976 Am J Respir Cell Mol Biol, 2001
- The orphan receptor ALK7 and the Activin receptor  [ **ACVR2** ]  ALK4  [ **ACVR1B** ]  mediate signaling by Nodal proteins during vertebrate development.  Ref: 11485994 Genes Dev, 2001
- A recently cloned inhibin binding protein, InhBP p120, associates strongly with the type IB activin receptor  [ **ACVR2** ]  Alk4  [ **ACVR1B** ]  in a ligand responsive manner and interacts to a lesser extent with other activin and bone morphogenetic protein BMP type I and activin type II receptors.  Ref: 11266516 Mol Endocrinol, 2001
- When coexpressed with type I activin receptor  [ **ACVR2** ]  ActR I or ActR IB  [ **ACVR1B** ]  in concert with type II receptor ActR II  [ **ACVR2** ] , Smad7 efficiently reduced an early transcriptional response mediated by ActR I but had only a minimal effect on the response mediated by ActR IB  [ **ACVR1B** ] .  Ref: 10828018 Blood, 2000
- Crosslinking studies measuring binding of 125I activin A to the ALK4  [ **ACVR1B** ]  trunc mutants in the presence of ActRII  [ **ACVR2** ]  implicated the same residues.  Ref: 12665502 J Biol Chem< MedlineTA>J Biol Chem, 2000
- Surprisingly, activin had no effect on follistatin mRNA levels, despite the fact that FS D1h cells express ActRII  [ **ACVR2** ] , ActRIIB, and ALK 4  [ **ACVR1B** ]  ActRIB.  Ref: 12538636 Endocrinology, 2003
- RESULTS Activin A and receptors ActR I, ActR IB  [ **ACVR1B** ] , ActR II  [ **ACVR2** ]  are transcribed in corneal fibroblasts.  Ref: 11773015 Invest Ophthalmol Vis Sci, 2002
- Overexpression of wild type activin receptor  [ **ACVR2** ]  alk4  [ **ACVR1B** ]  1 restores activin antiproliferative effects in human pituitary tumor cells.  Ref: 12364468 J Clin Endocrinol Metab, 2002
- This effect was mediated, at least in part, by the type IB activin receptor  [ **ACVR2** ]  ALK4  [ **ACVR1B** ] .  Ref: 11034308 FEBS Lett, 2000
- The presence of inhibin subunits alpha, betaA and betaB, activin binding protein follistatin and activin receptors ActR I, ActR IB  [ **ACVR1B** ] , ActR II  [ **ACVR2** ]  and ActR IIB mRNA transcripts in bovine oocytes and preimplantation embryos was examined by reverse transcription polymerase chain reaction.  Ref: 11596877 Reprod Fertil Dev, 1998
- Analysis in native tissues from 12 normal thyroids, 16 goiters, and 5 Graves thyroids demonstrated mRNA expression of the activin subunit betaA, TGFbeta receptor type I and II, activin receptors type I receptors alk2 and alk4  [ **ACVR1B** ] , and activin type II receptors actRII  [ **ACVR2** ]  and actRIIb.  Ref: 11069203 Horm Metab Res, 2000
- The expression of both type I ALK 4  [ **ACVR1B** ]  ActRIB and type II ActRII  [ **ACVR2** ]  receptors for activin was detected in F5 5.fl cells.  Ref: 11078996 Eur J Endocrinol, 2000
- These affinities are similar to those for binding to a chimeric receptor in which the ECD1 of CRFR2beta replaces the ECD of the type 1B activin receptor  [ **ACVR2** ]  ALK4  [ **ACVR1B** ] .  Ref: 12611895 J Biol Chem, 2003
- In this study, we found the expression of activin type I receptors ActR I and ActR IB  [ **ACVR1B** ]  and type II receptor ActR II  [ **ACVR2** ]  on human fetal lung fibroblasts HFL 1 by RT PCR and immunocytochemistry.  Ref: 11261590 Life Sci, 2000
- SB 431542 is a potent and specific inhibitor of transforming growth factor beta superfamily type I activin receptor  [ **ACVR2** ]  like kinase ALK receptors ALK4  [ **ACVR1B** ] , ALK5, and ALK7.  Ref: 12065756 Mol Pharmacol, 2002
